# A rare bacterial infection of the gastrointestinal tract: *Clostridium ventriculi*

**DOI:** 10.4322/acr.2024.469

**Published:** 2024-02-01

**Authors:** Sonali Mishra, Ashok Singh, Arvind Kumar, Ravi Hari Phulware

**Affiliations:** 1 All India Institute of Medical Sciences, Department of Pathology & Laboratory Medicine, Rishikesh, Uttarakhand, India

**Keywords:** Clostridium ventriculi, Sarcina ventriculi, Bacteremia, Helicobacter pylori, Gastroparesis

## TO THE EDITOR

In 1942, John Goodsir,^1^ a Scottish anatomist and biologist, first described *Sarcina ventriculi* after the microscopic analysis of the gastric contents of a patient with daily vomiting, abdominal pain, and stomach ulcer suspicion. *Sarcina ventriculi* is an obligate anaerobe gram-positive coccus with carbohydrate fermentative metabolism.^2^ In 1994, phylogenetic analyses (16 rRNA gene sequences) revealed that *S ventriculi* belonged to the genus Clostidium.^3^ However, in 2016, the genus *S ventriculi* to *Clostridium ventriculi* was proposed.^4^ The bacteria can survive in varied conditions, including low stomach pH.^5^ They are found in cereal seeds, soil, mud, and the stomachs of humans, rabbits, and guinea pigs.^4^ The natural habitat of Sarcina is the soil, and infection is contracted in humans and animals by ingesting food contaminated with soil particles.^6^ Primarily as a disease of the ruminants, its incidence in humans in the 19^th^ century was very low; hence, the pathogenicity is not fully understood.^5-6^ Since 2010, however, a sudden rise in cases occurred, consistent with a reappearance in humans. This may be due to changes in feeding habits, food hygiene, and food quality or bacterial selection.^4-5^ Lam-Himlin et al.^5^ also suggested that patients with *C. ventriculi* infection and pre-existing gastric mucosal defects are more susceptible to life-threatening outcomes, and the bacteria don’t directly invade the gastric wall.

In humans, *Clostridium ventriculi* causes a disease that mainly affects adults; however, patients ranging from 3-73 years have been reported. It is more common in females than in males.^5-6^ The spectrum of clinical features of *Clostridium ventriculi* infection in humans is broad, ranging from asymptomatic patients with incidental findings of the organism to patients presenting with acute abdomen due to gastric perforation.^5-6^ The most recurring symptom is delayed gastric emptying and/or gastric outlet obstruction. Patients also present with nausea, a characteristic “sarcinous vomit” (intractable, frothy vomit in patients with chronic disease of the stomach), and abdominal pain.^2-5^ Tim G. J. de Meij et al.^2^ reported Sarcina-related ulcerative gastritis and esophagitis in children with hematemesis. There are case reports of co-occurrence of Sarcina with Helicobacter pylori, Giardia, or in a background of cystic fibrosis, coeliac disease, or upper gastrointestinal surgery.^4-6^

**Figure 1:**
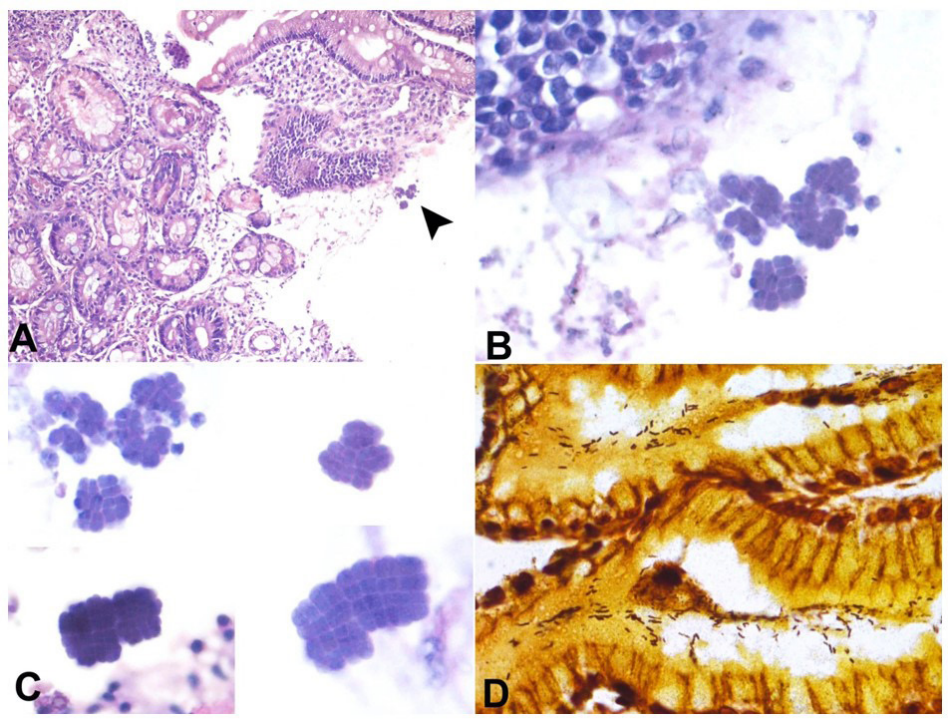
**A** - Photomicrograph from gastric biopsy shows gastric glands with intermixed chronic inflammatory cell infiltrate and presence of bacterial clumps (H&E, 100X); **B** - Higher magnification shows the presence of Clostridium ventriculi (H&E, 400X); **C** - Photomicrograph shows different forms of Clostridium ventriculi like tetramers, octamers (H&E, 1000X); **D** - Special stain for Warthin-starry highlights the Helicobacter pylori co-infection (400X).

Figure 1 refers to a 56-year-old male patient who presented to the gastroenterology outpatient department complaining of delayed gastric emptying, nausea, vomiting, abdominal pain, and early satiety. The upper gastrointestinal endoscopy revealed Los Angeles grade D (LA-D) reflux esophagitis, where mucosal breaks involved more than 75% of the esophageal mucosal circumference. Pyloric stricture was also suspected of a neoplastic origin in correlation with clinical and radiological findings.

Biopsy was taken from the pyloric stricture segment. Histopathological examination was done on the formalin-fixed paraffin-embedded sections stained with hematoxylin and eosin. This showed multiple gastric mucosal bits showing a *Clostridium ventriculi*-like organism. Few glands showed activity, i.e., the presence of neutrophils in the epithelium, and the lamina propria had dense acute inflammatory infiltrate with prominence of predominantly eosinophils and few neutrophils. Many packets of Clostridium ventriculi were interspersed in the lamina propria and along the luminal surface of the glands. A Warthin starry stain was done that showed co-infection by H. pylori.

Morphologically, they are nearly spherical and arranged in characteristic packets, forming tetrads or packs of eight or more. These packets are formed due to division in perpendicular planes. The size of individual spherules ranges from 1.8 to 3um. These spherules are refractory, basophilic on hematoxylin and eosin, and show wall flattening between the cells.^5-6^

There is no protocol defined for the treatment of *Clostridium ventriculi*. If it is isolated in a patient with dysphagia or substernal burning, a combination of proton pump inhibitors and prokinetic therapy is recommended. Eradication with antibiotics is advised if the patient has esophageal or gastrointestinal mucosa ulceration to avoid the risk of perforation.^4-6^ Any route for antibiotics may be used. Fasting seems to cut off the carbohydrate source of the bacteria, thus contributing to clinical improvement.^1,2.5^ To conclude, given the growing incidences of Sarcina infection and the possibility of life-threatening complications, timely recognition must be done, and infection treatment must be initiated.

## References

[1] Goodsir J, Wilson G (1842). History of a case in which a fluid periodically ejected from the stomach contained vegetable organisms of an undescribed form. Edinb Med Surg J.

[2] de Meij TGJ, van Wijk MP, Mookhoek A, Budding AE (2017). Ulcerative gastritis and esophagitis in two children with *Sarcina ventriculi* infection. Front Med (Lausanne).

[3] Willems A, Collins MD (1994). Phylogenetic placement of Sarcina ventriculi and Sarcina maxima within group I Clostridium, a possible problem for future revision of the genus Clostridium. Request for an opinion. Int J Syst Bacteriol.

[4] Lawson PA, Rainey FA (2016). Proposal to restrict the genus Clostridium Prazmowski to Clostridium butyricum and related species. Int J Syst Evol Microbiol.

[5] Lam-Himlin D, Tsiatis AC, Montgomery E (2011). Sarcina organisms in the gastrointestinal tract: a clinicopathologic and molecular study. Am J Surg Pathol.

[6] Al Rasheed MR, Senseng CG (2016). Sarcina ventriculi: review of the literature. Arch Pathol Lab Med.

